# Do patients receive recommended treatment of osteoporosis following hip fracture in primary care?

**DOI:** 10.1186/1471-2296-7-31

**Published:** 2006-05-09

**Authors:** Robert J Petrella, Tim J Jones

**Affiliations:** 1 Department of Family Medicine and Department of Physical Medicine and Rehabilitation, Schulich School of Medicine, Faculty of Medicine and Dentistry, 1490 Richmond St London, Ontario, N6G 2M3, Canada; 2 School of Kinesiology, Faculty of Health Sciences, University of Western Ontario, 1490 Richmond St, London, Ontario, N6G 2M3, Canada

## Abstract

**Background:**

Osteoporosis results in fractures and treatment of osteoporosis has been shown to reduce risk of fracture particularly in those who have had a history of fracture.

**Methods:**

A prospective study was conducted using patients admitted to a hip fracture rehabilitation program at a large referral center to evaluate the use of treatments recommended for secondary prevention of osteoporotic fracture between September 1, 2001 and September 30, 2003. The frequency of medication use for the treatment of osteoporosis including estrogen replacement therapy, bisphosponates, calcitonin, calcium and vitamin D therapy was determined on admission, at 6 weeks post discharge and one year following discharge. All patients were discharged to the care of their family physician. All family physicians in the referral region received a copy of the Canadian Consensus recommendations for osteoporosis management 1–3 months prior to the study.

**Results:**

During the study period, 174 patients were enrolled and 121 completed all assessments. Fifty-seven family physicians were identified as caring for 1 or more of the study patients. Only 7 patients had previous BMD, only 5 patients had previously been prescribed a bisphosponate and 14 patients were taking calcium and/or vitamin D. All patients were prescribed 2500 mg calcium, 400 IU vitamin D and 5 mg residronate daily during rehabilitation and at discharge.

Following discharge, a significant improvement was seen in all clinical indices of functional mobility, including the functional independence measure (FIM), walking distance, fear of falling score (FFS), and the Berg balance score (BBS). At six weeks a significant (p < 0.01) decrease in calcium and vitamin D use was observed. All patients remained compliant with residronate therapy. At twelve months 71 patients remained on residronate (p < 0.01), 10 were now taking alternate bisphosphonate therapy and few were taking calcium and/or vitamin D (p < 0.001). FIM, FFS and Berg scores were significantly decreased from discharge (p < 0.001) while walking distance was unchanged.

**Conclusion:**

Few patients admitted for hip fracture had previously taken recommended osteoporosis therapy including bisphosphonates. While compliance with Canadian Consensus recommendations was observed at six weeks, this was not the case at twelve months post hip fracture rehabilitation. Interventions to improve not only the detection and treatment of osteoporosis but also the ongoing treatment and management post-fracture need to be developed and implemented.

## Background

Osteoporosis is a chronic and progressive condition that leads to decreased bone mass and skeletal fragility; in turn, these conditions can lead to fractures, disability, pain and even death. [1-5]

The lifetime risk for a typical osteoporotic fracture (for example of the wrist, hip or vertebrae) is about 40%. [1-4] The annual cost of treatment of osteoporosis and its sequela in the United States is estimated about $13.8 billion dollars [1,3-5] compared with $7.5 billion dollars for congestive heart failure and $6.2 billion for asthma[6].

Ideally, osteoporosis should be prevented before bone mass is lost or fractures occur. Nevertheless, an important complimentary strategy is to identify patients who already have had a typical osteoporotic fracture and institute treatments aimed a further secondary prevention. [4-7] In post menopausal women, at least 80%–90% of fractures of the wrist, hip or vertebrae are associated with osteoporosis, [8-10] and a patient with both osteoporosis and a fracture has approximately a twenty-fold risk of future fracture compared with a patient who has neither osteoporosis nor a history of fracture.[2,9,11] Furthermore, the risk of recurrent fracture begins to rise within the first months and year of the index fracture event.[12] Because patients with osteoporosis are at such high risk for recurrent fracture, they may also derive the greatest absolute benefits for treatment. Since the early 1990's, physicians have had several well-tolerated effective therapeutic options that are described in consensus recommendations including bisphosphonates, calcitonin and estrogen.[1,2,6,7,13] These agents have been shown to increase bone mineral density, and the relative reduction in risk for fracture with each treatment is about 40 to 60%.[1,2,6,7,13] Moreover, the benefits of treatment extend to all available subgroups of patients including the elderly, those with multiple previous fractures, and those with the lowest bone density[14].

Despite the relative ease with which high risk patients with symptomatic fracture can be identified, and given the availability of effective treatments, some recent studies have suggested that physicians may be missing important opportunities for secondary prevention, particularly for patients with non-vertebral fractures.[4,11,15-20] For example, the rate of initiation of new osteoporosis treatment has been reported between five and sixteen percent following a wrist fracture,[4,11,15-17] one to nine percent following hip fractures,[15,18,19] and eighteen to thirty-nine percent following symptomatic vertebral fractures.[15,20] Indeed, Andrade et al [20] found that most women who had experienced a fracture of the hip, vertebra or wrist did not receive drug treatment for osteoporosis even up to one year following the fracture. These previous studies were not population based;[11,16,18-20] were not restricted to postmenopausal women;[11,15,16,18,19] did not look at inpatient groups who were easy to identify and had a short duration of followup.[4,18,20] Therefore the existing studies are somewhat limited.

Hence, we undertook the present study in an opportunistic cohort of inpatient hip fracture patients at a musculoskeletal (MSK) rehabilitation referral center to document the rate of osteoporosis treatment at the time of fracture and during the year following fracture to examine correlates of receiving treatment after fracture. We also supplemented this strategy with a family physician brief educational intervention to expose them to the Canadian Consensus recommendations [2] and also obtain information regarding their perception of confidence with treating and preventing osteoporosis in fractures during this study. We hypothesized that our findings might expose potential important but missed opportunities for treatment in the post hip fracture setting or secondary prevention setting.

## Methods

We conducted a prospective study among patients admitted for hip fracture rehabilitation in the MSK program at Parkwood Hospital, London, Ontario (pop ~400,000). Both men and women who were admitted between three and seven days post fracture for a three to four week length of stay rehabilitation program were enrolled between September 1, 2001 and September 30, 2003. Informed consent was obtained by the study team and the study was approved by the University of Western Ontario Review Board. We excluded patients who had elective arthroplasty or had multiple trauma beyond the single hip fracture, had diagnosed cancer or concurrent medical problems requiring aggressive intervention. The date of the initial fracture during the study period was identified while the date of discharge was used as the index for followup at six weeks and twelve months.

We reviewed medical histories of the sample on admission for their ability to fulfill the selection criteria as well as obtaining a history of current medical problems, concomitant medications, BMI, current functional assessment using the functional independence measure (FIM) [21], the Berg balance score (BBS) [22], fear of falling score (FFS) [23] and walking distance. We also determined whether previous osteoporosis treatment was received including bisphosphonate therapy, calcium and Vitamin D, and whether a previous BMD had been done. The inpatient MSK rehabilitation program has been previously described [24]. In brief, all patients received combination of occupational and physical therapy at least three times per week for two one hour periods per session.

Prior to the study, a brief educational intervention was delivered by mail to all practicing family physicians within the referral network of Parkwood Hospital comprised of a statement of current Canadian Consensus recommendations for osteoporosis management [2]. We determined physician confidence and perception of knowledge of osteoporosis management using a ten point visual analog score (VAS) with anchors from 0 (no confidence or no knowledge) to 10 (completely confident or completely knowledgeable).

All patients were prescribed calcium (1250 mg daily), vitamin D (400 IU daily) and residronate (5 mg daily) during rehabilitation and at discharge. Patients were screened at entry for evidence of Vit D deficiency.

Adverse events were reported and recorded at each measurement period (admission, discharge, 6 weeks and 12 months followup). We also recorded the type of gait device being used on discharge as well as whether DVT prophylaxis was prescribed.

The overall frequency of use of osteoporosis therapy during the year following the date of discharge from the MSK rehabilitation program were used to estimate compliance with current recommendations. A BMD was also done during the inpatient period and at 12 month followup. Statistical significance of differences was tested using Pearson χ^2 ^statistics and Mantel-Haenszel test for linear association. Logistic regression was used to estimate the strength of the association between patient characteristics and the use of treatments for osteoporosis following discharge.

## Results

We identified 174 patients (78% female) with a mean age of 83 ± 10 years (Additional file 1) who were diagnosed and treated for a fracture of the hip during the study enrolment and followed for one-year post discharge. Fifty-seven family physicians were identified as primary care providers for the patient cohort. All physicians had received the Canadian Consensus recommendations and completed the two VAS questions prior to the study. Patients enrolled in the study represented approximately 66% of all admissions to the MSK rehabilitation program during the study period. Reason for exclusion from the protocol include refusal to consent, documented intolerance to bisphosponate therapy, multiple trauma, concurrent medical illness requiring intensive therapy or cancer. On admission to the MSK rehabilitation program, 33 women used estrogen therapy, only 14 were using calcium and/or Vitamin D therapy and only 5 had been using a bisphosphonate medication. None were using calcitonin therapy and no men were using calcium, vitamin D or bisphosphonate. Only 7 patients had a previous BMD. The BMD score on admission to rehabilitation was significantly lower among women than men (p < 0.05). The average FFS was 14 ± 1.3 and all patients reported at least one previous fall resulting in trauma. Patients were cognitively intact (having a mini mental status score of 27 ± 1.5. Twenty-seven patients had a previous documented fracture while 6 patients had evidence of vertebral osteoporotic fracture. Increasing age was associated with a significant (p < 0.05) decrease in previous osteoporosis treatment or absence of previous BMD. Similarly, increasing age was associated with higher numbers of co-morbid diagnoses and the number of concomitant medications. The most common diagnosis was that of hypertension (39%). Those patients with a previous history of fracture or osteoporosis treatment had higher scores of fear of falling and greater number of previous significant falls.

No adverse events were recorded during the rehabilitation period. One-hundred and six patients were discharged on DVT prophylaxis with instructions to maintain this prophylaxis for a total of six weeks post fracture. Mean length of rehabilitation stay was 3.6 weeks ± 2.3 weeks. Bone mineral density scores during the inpatient period revealed all patients to have femoral neck osteoporosis in the non-fracture site. One-hundred and twenty-seven patients were discharged as full weight bearing on the affected hip while remaining patients were at least 75% weight bearing. All patients were prescribed a gait aid on discharge. The FFS significantly decreased from admission (p < 0.001), while walking distance (p < 0.05), FIM (p < 0.01) and BBS (p < 0.001) were all significantly increased (Additional file 1).

At 6 weeks post discharge all patients returned for followup assessment. Only 16 patients regularly used a prescribed gait aid. We observed a significant decrease in Vitamin D (p < 0.001) and calcium (p < 0.01) compared to discharge (Additional file 1). All patients remained compliant with residronate therapy. None of the patients were using DVT prophylaxis therapy. Fear of falling score, FIM, and BBS were similar to discharge (p > 0.05) while walking distance was significantly decreased from discharge (p < 0.05). All patients were seen by their family physician by 6 weeks followup. All family physicians had competed pre-study Canadian Consensus detailing and VAS perceived confidence and knowledge questions. Perceived osteoporosis management confidence was 9.4 ± 0.9 while perceived knowledge of management recommendations was 7.9 ± 1.3.

At twelve months followup, 71 patients remained on residronate, and 10 were taking alternate bisphosphonate (Additional file 1). Reasons for discontinuing bisphosphonate included a lack of perceived efficacy, non-specific side-effects, and constitutional symptoms including constipation. Fear of falling score had significantly decreased from discharge toward admission score (p < 0.05), while FIM (100) and BBS (39) had also decreased back to discharge scores but these changes were not statistically significant. Walking distance was unchanged from the 6 week distance but significantly reduced from discharge (p < 0.01). Only eight patients regularly used a gait aid at twelve months but only two of these were prescribed. Bone mineral density scores showed no significant change from discharge among participants who had discontinued bisphosphonate. Those who remained on a bisphosphonate significantly improved their femoral neck BMD score (p < 0.05) (Additional file 1). Only 20 physicians recalled the Canadian Consensus recommendations and felt these materials assisted their osteoporosis management. Surprisingly, perceived confidence and knowledge VAS scores were unchanged (8.9 and 9.5 respectively) for the group from discharge (Additional file 1).

Additional file 2 presents the odds ratio (OR) estimates and 95 % confidence intervals (CIs) for the association of hip fracture, patient age, number of co-morbidities and FFS with the use of osteoporosis recommended treatment at twelve months. Compared with those who were older, those patients who were < 70 years of age were more likely to continue osteoporosis treatment at twelve months. Further, those patients with a higher FFS and greater number of co-morbidities tended to continue osteoporosis recommended therapy at twelve months.

## Discussion

In a large representative cohort of patients admitted for rehabilitation following a hip fracture very few previously had a BMD or received recommended osteoporosis therapy. Admission for rehabilitation was associated with high scores for fear of falling and previous fall behavior. While all patients continued recommended bisphosphonate therapy at six weeks, most had discontinued calcium and vitamin D. Further, most had discontinued bisphosphonate by 12 months while continued calcium or vitamin D. This was despite documented osteoporosis and followup with their family physician who expressed a high degree of perceived confidence and knowledge regarding osteoporosis management recommendations.

Patients who were taking osteoporosis therapy or had previous BMD on admission tended to be younger, have less fear of falling and were more functionally independent. Further, being younger, having less fear of falling and being more functionally independent was associated with higher likelihood of remaining on recommended therapy at 12 months post discharge after hip fracture. Conversely, patients at higher risk for functional dependence in terms of co-morbidities, fear of falling, previous fall history, and FIM were less likely to have been on recommended osteoporosis therapy or have had a BMD at the time of fracture, and were less likely to continue osteoporosis recommended therapy at twelve months. Age as a risk factor for inadequate treatment has been previously documented for many conditions [14,25], including osteoporosis.[4,16,20]. Nonetheless, given the fact that osteoporosis is, itself, an age related condition and that increasing age is a powerful independent risk factor for future fracture as well as a second fracture, we might have expected that elderly patients would be more likely, and not less likely, to receive treatment than their younger patients who had lower risk. A 75-year-old woman has, on average, a life expectancy of twelve years [14] and the benefits of osteoporosis treatment, in terms of increasing bone marrow density and decreased fracture risk are seen within a year [16,26]. Certainly, Ensrud et al [10] has suggested that it may never be too late, in life or in the disease process, to prevent fractures with appropriate treatment.

Overall, our results are concordant with those in previous studies [4,11,15-20]. The problem of under treatment of osteoporosis in patients with symptomatic fracture has been documented using different methods, across different populations, and at different health care delivery systems. Certainly in our cohort there was no problem with access to treatment and all patients had a family physician who were not only confident in their ability to treat osteoporosis but they also received a brief educational intervention regarding osteoporosis recommendations. Further, all patients were prescribed osteoporosis therapy during and following discharge from the MSK rehabilitation program. It is interesting therefore, that under treatment was so prevalent. Although many barriers to optimal treatment may exist, one of the major contributors to this problem may be at the level of the health care delivery system. This seems to be corroborated by our results. There seems to be a clinical disconnection between physicians responsible for treating symptomatic fractures, and the primary care physicians who are responsible for the detection and ongoing management of osteoporosis [4,16]. We believe that any health care system that does not explicitly provide the means to link acute and primary care providers will be at risk for delivering sub-optimal osteoporosis care.

In addition, an element of clinical inertia may be present as shown in previous studies.[20] Clinical inertia or the failure of the health care providers to initiate or change treatment when the health status of a patient indicates that such a condition is necessary, has been described for several other chronic medical conditions including diabetes, hypertension, and hyperlipidemia.[17,18,27,28] Further, patients may have misconceptions regarding the importance of ongoing treatment of chronic diseases.[29] Thus recommended strategies to avoid clinical inertia have included the provision of various forms of education; systematic, targeted reminders and feedback from practice performance; and the development of consensus guidelines to address important quality of care problems in these clinical conditions.[18] These implementation systems should be explored further in primary care settings.

There may have been a view among family physicians that insufficient evidence existed about the optimal management of osteoporotic fractures. However, we provided the most recent Canadian Consensus recommendations^(2) ^and a clear statement that was emphasized within these recommendations regarding appropriate prevention and therapeutic measures in particular with calcium, Vitamin D, and bisphosphonates.

The major strength of this prospective study was the longitudinal design over 12 months and the linked access of the rehabilitation setting to the attending primary care physicians. Further, our ability to address the perception of knowledge regarding osteoporosis treatment and prevention was enhanced through direct survey of the attending physicians within our patient cohort. We provided not only the recommendations for osteoporosis prevention and management but provided discharge prescriptions of these medications prior to the patients attending to their family physician. These strategies should have facilitated management of these patients at 12 months. However, it is clear that more structured implementation systems and followup are required. We also collected corroborative outcome measures related to patient risk of future falls, fracture and disability. We were surprised to see those patients at higher rather than lower risk were more likely not to receive recommended osteoporosis therapy.

Some limitations should also be noted. Inclusion of a more comprehensive osteoporosis management tool with recent supportive studies may have maintained higher rates of osteoporosis management among these patients. Certainly Consensus Guidelines are only guidelines and are based on the best available evidence at that time but some clinicians may prefer to weigh the evidence themselves despite knowledge of guidelines as seen in other therapeutic areas. [30] Conversely, addition of more clinical data may been an overburden and may have further diluted the minimal educational intervention we used and produced even poorer results. It would have been interesting to see what the standard of care among physicians was for other patients with osteoporosis not admitted for fracture. Further, inclusion of a comparator group (ie. no osteoporosis) or condition (ie. hypertension) may have provided insight regarding more systemic issues of chronic disease management in family practice.

Given the heterogeneity of our study population we were able to include both men and women who had suffered an osteoporotic fracture however few men were admitted during the study period. The age diversity also allowed us to understand some of the present gaps in treatment related to age. One of our assumptions was that estrogen therapy was likely prescribed for osteoporosis treatment as its efficacy in terms of cardiovascular protection was an issue that was clearly described during the course of our study. However, it is also plausible that women may have started their estrogen therapy prior to the disclosure of this new information and continued on it despite the evidence. Other patients may have been on estrogen therapy because of menopausal symptoms. However, very few patients took estrogen treatment at admission and did not alter this over 12 months such that this aspect of osteoporosis therapy was unlikely to have affected our results.

## Conclusion

In conclusion, we found that the vast majority of patients who had suffered an osteoporotic fracture were not taking recommended therapy at admission or 12 months post discharge from a rehabilitation program despite prescription of recommended osteoporosis therapy at discharge and the use of an educational intervention for family physicians. These findings were despite improved BMD at 12 months among those who did adhere to prescribed therapy at discharge and high levels of perceived confidence and knowledge regarding osteoporosis therapy among their family physicians. Older age, increased fear of falling and impaired functional independence and mobility seemed to be related to lower likelihood of patients being maintained on osteoporosis treatment at 12 months. Our findings suggest an opportunity to improve the quality of osteoporosis care in family practice. It is unlikely that publication of consensus recommendations or didactic dissemination of clinical practice guidelines alone are or will be sufficient to improve osteoporosis management in patients at high risk for recurrent fractures and morbidity. Certainly to close this care gap and improve the quality of care for patients with osteoporotic fractures, we believe that innovative, multi-faceted interventions need to be developed and implemented in cooperation with the health care system, primary care, rehabilitation physicians and patients.

## Competing interests

R Petrella declares no competing interests.

T Jones was an employee of Proctor and Gamble at the time of publication of this study.

## Authors' contributions

R Petrella contributed to the concept, design, data collection, analysis and writing of the manuscript.

T Jones contributed to the design and data collection of the study.

Both authors approved the final manuscript.

## Pre-publication history

The pre-publication history for this paper can be accessed here:



## Supplementary Material

Additional File 1 Table 1. Osteoporosis Table 1. Study subject characteristics. Click here for file

Additional File 2 Table 2. Osteoporosis Table Petrella. Patient characteristics and association with persistence to treatment. Click here for file
