# Supplementary material for: Do patients receive recommended treatment of osteoporosis following hip fracture in primary care?
Source: BMC Fam Pract. 2006 May 9;7:31. doi: 10.1186/1471-2296-7-31 (PMC1524774; doi:10.1186/1471-2296-7-31)
Supplement: Additional File 1 — Table 1. Osteoporosis Table 1. Study subject characteristics. [file 1471-2296-7-31-S1.doc]

**Table 1. Characteristics of study population.**

**Admission Discharge 6 weeks 12 months**

N=174 N=174 N=174 N=121

| Age (years) | 83 ± 10 |  |  |  |
| --- | --- | --- | --- | --- |
| Gender (female) % | 78 |  |  | 80 |
| BMI (kg ·m^-1^) | 24 ± 3 |  |  | 23 ± 5 |
| BMD (femoral)  T score | 3.38 ± 1.77 |  |  | 3.17 ± 1.19 |
| BMD (femoral)  (those compliant with bisphosponate at 12 months (N=71) | 3.41 ± 1.62 |  |  | 2.62 ± 1.11^*^ |
| Calcium use  ≥ 2500mg daily  (# patients) | 14 | 174^+^ | 110^+^ | 5^*^ |
| Vitamin D use  ≥ 400IU daily  (# patients) | 14 | 174^+^ | 125^+^ | 31^**^ |
| Bisphosphonate use  (# patients) | 5 | 174^+^ | 174^+^ | 81^+^ |
| FFS | 14 ± 3 | 11 ± 3^*^ | 13 ± 2 | 15 ± 1 |
| FIM | 83 ± 7 | 108 ± 9^**^ | 96 ± 14^*^ | 91 ± 8 |
| BBS | 29 ± 11 | 52 ± 8^+^ | 45 ± 10^**^ | 38 ± 7^*^ |
| Total walking distance test (meters) | 383 ± 25 | 1383 ± 574^+^ | 912 ± 613^*^ | 768 ± 588^*^ |
| Physician Confidence (VAS-cm) |  |  | 9.4 ± 0.7 | 9.5 ± 0.5 |
| Physician Knowledge (VAS-cm) |  |  | 7.9 ± 1.3 | 8.9 ± 1.1 |

BMI= body mass index; BMD= bone mineral density; FFS=fear of falling score^23^; FIM=functional independence measure^21^; BBS=Berg balance score^22^

* p<0.05; ** p<0.01; + p<0.001
