# Supplementary material for: Do patients receive recommended treatment of osteoporosis following hip fracture in primary care?
Source: BMC Fam Pract. 2006 May 9;7:31. doi: 10.1186/1471-2296-7-31 (PMC1524774; doi:10.1186/1471-2296-7-31)
Supplement: Additional File 2 — Table 2. Osteoporosis Table Petrella. Patient characteristics and association with persistence to treatment. [file 1471-2296-7-31-S2.doc]

|  |  | **Population**  **(N = 121)** |
| --- | --- | --- |
| Age, y |  |  |
| 60 to < 65 |  | 1.08 (.082-1.27) |
| 65 to < 70 |  | 1.02 (0.76-1.36) |
| 70 to < 75 |  | 0.88 (0.51-0.90) |
| 75 to < 80 |  | 0.72 (0.40-0.73) |
| ≥80 |  | 0.28 (0.21-0.38) |
| Number of co-morbidities |  |  |
| None |  | 1.0 (Reference) |
| 1-2 |  | 1.39 (1.18-1.65) |
| 2-5 |  | 1.09 (0.77-1.55) |
| >5 |  | 0.78 (0.52-1.01) |
| Fear of falling score |  |  |
| <10 |  | 1.09 (1.01-1.53) |
| 11-12 |  | 1.0 (Reference) |
| 12-14 |  | 1.36 (1.07-1.72) |
| 14-16 |  | 1.43 (1.12-1.82) |

Table 2. Associations Between Patient Characteristics and Persistence with Recommended Osteoporosis Treatment in the 12 Months Following Fracture of the Hip

**Odds Ratio (95% CI)**
